# Workers’ access to Swedish opera houses and concert halls, 1898–2019

**DOI:** 10.1007/s10824-021-09437-0

**Published:** 2021-11-29

**Authors:** Staffan Albinsson

**Affiliations:** School of Business, Economics and Law, University of Gothenburg, Gothenburg, Sweden

**Keywords:** Social inclusion, Ticket pricing, Performing arts, Cultural economics, N34, J31, D12, H42

## Abstract

In this study ticket prices to Swedish opera houses and symphony orchestra concerts are compared to wages during the 1898–2019 period. Both wages and ticket prices have increased continuously. The same kind of policy objectives concerning social inclusion of disadvantaged groups that were established in the beginning of the twentieth century is still proclaimed. The most favourable ticket pricing policies for buyers were used in the decades around the first national Cultural Policy Act from 1974. The study shows that ticket price levels have risen thereafter to a level much less favourable for low-income workers. Managements do use some price discrimination tactics. However, they do it uniformly for all events. They now focus on the promotion of special, ‘popular music’-based events as a response to social inclusion directives. The idea is that attending such performances will make visitors interested in the normal repertoire, as well. The choice of high-level ticket prices for the traditional content means that the standard audience remains monocultural.

## Introduction

In a previous article the author presented the takeover from the aristocracy of the new bourgeoisie as box tenants in European opera houses after the French Revolution. Here yet another step forward in history is taken. The focus is on the twentieth century cultural policy endeavour for inclusion in Swedish opera houses and concert halls of the underprivileged and disadvantaged. In the previous article a prediction was made:

It is much less likely that the growth of workers’ incomes during the twentieth century has led to a similar increase in working class demand for opera as it did for the bourgeoisie during the period studied. . . . If opera demand increases in the near future, this will possibly be based on the growth of income for population segments that already constitute opera audiences. (Albinsson, 2020).

This idea is elaborated here. Workers’ wages are compared to clerks’ salaries and with ticket prices. The study is longitudinal. It includes data from the opening of the Oscarian opera house in Stockholm in 1898 until 2019. The main research question is the following: Does the explicit cultural policy goal for social inclusion mean that increases in ticket prices have mirrored raised wages levels for workers? If not: has this been detrimental to workers’ access to opera houses and concert halls? An important sub-issue is whether price discrimination policies have been implemented to the advantage of the low-income strata of society. The choice of Sweden is principally due to the long tradition of explicit political goals to include, first, ‘manual workers’ (Engberg, 1921), then ‘the disadvantaged’ (SOU, 1972:66, 155), ‘underprivileged groups’ (SOU, 1972:66, 158), ‘an open social community’ (SOU, 2009:16, 21) and, now, ‘the broad public’ (Uppsala, 2020, 5; Region Västra Götaland, 2019, 13). Furthermore, because the work was undertaken during the Covid-19 pandemic, the author was restricted to work within the borders of his country of residence.

Modern Swedish cultural policy is based on the ideal of *folkbildning*, which was inspired by the Humboldtian concept of *Bildung* (Hofmann, 2010). In Sweden, *folkbildning* is ascribed to the mostly voluntary education and formation of unprivileged groups of the population that was established within the framework of the three social movements that emerged during the nineteenth century: the free church movement, the sobriety movement and the labour movement (Key, 1897; SOU, 1952:23, 167; SOU, 1972:66, 47–51; Sundgren, 2007, ch. 1; Proposition, 2009/10:3, 111; Folkbildningsrådet, 2020).

The major Swedish opera houses and concert halls are state, regional or municipal government institutions. Although they are all separate entities outside the governmental administration, their boards consist of politically nominated members. Furthermore, their funding is largely tax based. National government institutions receive funding only from the *Riksdag* (the national parliament). Regional institutions are funded by both the *Riksdag* and the regional cultural councils. Municipal institutions normally receive funding from all three levels of governments. While the first CPA of 1974 was more or less copied word for word into regional and municipal CPAs, the 2009 national act has been reformulated into regional and municipal cultural strategies with more concrete objectives than the national cultural policy goals. Most of the latter start with the rather undecisive Swedish word *främja*, which translates into ‘encourage, promote, further, or facilitate’, of which ‘promote everyone’s opportunities for cultural experiences, *Bildung*, and develop their creative abilities’ is the most relevant for this study. The initial *folkbildning* ideal is still an inspirational source in the CPAs on all three levels. The inclusion of all inhabitants in cultural activities is part and parcel of all the CPAs. A major change has also occurred in Sweden with the broadening of what is regarded as, in this case, music of high quality. However, the funding of what were formerly considered to be culturally worthy fine arts institutions has not diminished.

The *Socialdemokratiska Arbetarpartiet* (SAP; Social Democratic Workers’ Party) was formed in 1889. From its perspective, the role of cultural policy was to improve the cultural standard for underprivileged sections of the population and thus, particularly, to elevate the working class to a more refined level where it could be considered worthy of participating in the country’s political affairs.

For the working class the conquest of culture became a way of qualifying for the position of political power it sought. The task was to foster the taste of the working class and to make it rise to ever higher cultural levels. This taste-formation attitude is both strong and self-evident in the whole of society even in the 1940s. (Sundgren, 2007, 272)^1^

From a near majority parliament position the SAP led the development of the Swedish welfare society from 1932 to 1976.

The 1947 parliamentary Music Inquiry pointed out that the Swedish music arena by then was more extensive than ever before. Although the working class, like others, thereby gained improved accessibility to high-quality music experiences, the audience still consisted mainly of the better off. Ticket prices were identified as an obstacle: ‘The cost of concert attendance certainly seems to, largely, restrain the participation at concerts of a large share of the population in the desired extent’ (SOU 1954: 2, 109–110).

In 1921 Arthur Engberg, a few years later the SAP Minister of Education and Culture, wrote that ‘foxtrot and other nonsense’ should be worked against (Engberg, 1921). This tradition was followed by another SAP Minister of Education and Culture, Ragnar Edenman (1959), who worked for the cultural formation of the nation against the forces of ‘weekly tabloids, kitschy paintings, “schlager” music, films of dubious value and all kinds of “entertainment”’. The musicologist Martin Tegen (1949) discussed this issue along similar lines but with a musical twist: ‘The many layers of contemporary music audiences each have their own habits and needs, which are, occasionally, satisfied in a rather harsh, commercially calculating way, even in terms of such details as timbre and style.’

The overarching objective of the 1974 Cultural Policy Act/CPA was that it should ‘contribute to a better society with greater equality’. One of the eight sub-goals was: ‘cultural policy must to a greater extent be modelled with regard to the experiences and needs of disadvantaged groups’. Here one could expect that the working class could be, explicitly, considered as such a group and that low fees could be thought of as a way of framing the performing arts to its needs. However, there is no such consideration in the bill. Only ‘children, the disabled, institutionalised, immigrants and other ethnic groups as well as people in sparsely populated areas’ were regarded as disadvantaged (Proposition, 1974: 28, 300). Although the cultural policy bill was based on 38 years of SAP dominance, the decision on cultural policy was both broadly and deeply rooted in all parliamentary parties.

The 2004 Orchestra Inquiry’s report, ‘Professional orchestra music in Sweden’, identifies that also today there is a need for strategies and methods to reach a wider audience. The inquiry found that many orchestras were aware that the world around them had changed and, hence, new strategies had been drawn up. However, ‘these strategies have not been able to sufficiently counteract the decrease in audiences that has affected orchestral music.’ The inquiry states that ‘the audience today mainly consists of a Swedish [ethnicity] middle-class audience with a certain dominance of female visitors’. That is why the report results in, among other things, a proposal that a new directive should include conditions to ‘strengthen the diffusion of orchestral (and opera) music, but also concert music in other forms, to a broad audience’ (SOU, 2006: 34, 108–114).

A recent article by Wu et al. (2019) provides an extended cultural economics literature review. They find that ‘a positive effect of income and a negative effect of price on the demand for performing arts are mainstream results in cultural economics literature.’ Ziebe and O’Hagan (2013) make a similar argument, writing that ‘[p]rice and income are shown to be highly significant,’ but their research is based on data from individuals. Here, no such data are used. Tentative attempts with econometric tools using the average income levels for workers and clerks and the ticket price data collected here show a similar response. However, this study does not intend to establish such already known causality facts but rather tries to bring the pricing issue a small step further. Bearing the rather intuitive price-income relation in mind, the narrative is centred on how ticket prices in publicly owned or subsided opera houses and concert halls have (or have not), over time, facilitated low-income earners’ attendance in line with public funders’ cultural policy ambitions that date back more than a century.

The ticket pricing issue has been little researched in Sweden. In the USA, where opera houses and symphony orchestras are primarily based on private funding, the ticket price instrument is discussed in academic journals. Scheff (1999) discusses subscriptions versus single-ticket purchases. Rosen and Rosenfield (1997) discuss the economic principles in price discrimination on tickets. Cortey and Pagliero (2012) use a multiple regression model to account for a multitude of artists, venues, cities and concert characteristics. They find that price discrimination through multiple ticket prices can increase revenues by 5%—the same result as Leslie (2004) finds in an earlier study of Broadway theatres. Eckard and Smith (2012) find a 4.2% revenue benefit from the use of multi-tier pricing at a major US pop music venue. Ekelund and Ritenour (1999) add the variable of time opportunity cost, adapting the approach introduced by Owen (1971) in their quantitative analyses on the demand for symphony orchestra tickets. Lange and Luksetich (1984) calculated the demand elasticities for 128 US symphony orchestras with respect to prices, quality and promotion. They found a negative price elasticity at a similar level (circa − 0.48) to what was found by other contemporary researchers (e.g. Withers, 1980). Ziebe (2009) discusses price elasticity in the demand for German theatre. She finds that the opportunity cost for leisure time has a higher negative elasticity factor than ticket price. The more people earn, the higher their ‘cost’ for participating in time-intensive performing arts consumption. The same pattern is found in this study of Swedish venues.

The main finding of the study is that workers now need to work for more hours to be able to pay for a ticket to an opera house or a concert hall than during the 1970s, despite the consideration of workers, explicitly or implicitly, as an ‘underprivileged group’ and their inclusion in the ‘broad public’ having been a major focal point in all national CPAs. Workers are compared to clerks because the latter are not generally considered to be underprivileged—the workers’ ‘penalty’ versus that of clerks is used in the analysis. Another finding is that opera houses do use elements of price discrimination strategies, whereas symphony orchestras and their concert halls engage less in this practice. However, most pricing strategies are uniform. They apply equally to all kinds of content. Instead publicly funded music institutions currently try to appeal to new audience groups through the use of repertoire from a fundamentally commercial musical domain in ‘special events’. The hope is that this will make such new listeners interested also in the standard repertoire and to ‘lower the venue thresholds’. The choice of a high level of ticket prices for the more traditional content means that this hope is in vain. The audience remains monocultural.

## The ‘folk’ concert and ‘folk’ performance

An early outlet of *folkbildning* ideals was the *Arbetarinstitut* (Workers’ Institute)—the first in Stockholm in 1880 followed by a second in Gothenburg three years later. The instigator, physician Anton Nyström, preferred to ‘prevent human suffering, weakness and crime rather than seek remedy for them once they have been created, and, further, that a large number of them owe their cause to inadequate upbringing, skewed education, prejudices, injustices, harmful pleasures and delights, drunkenness, etcetera.’ (Fredberg, 1919, 731). The idea of the workers’ institute was to spread information to all classes of society in the form of lecture series for a small fee at times when workers had the opportunity to attend. Beginning in 1895 the institute in Gothenburg promoted music through ‘folk’ concerts. The first series was sponsored by wealthy business and industry leaders. The Gothenburg institute lasted until 2015. During its first century it promoted more than 1600 ‘folk’ concerts (Berrman & Ideström, 1983, 94). It was most likely the last to use this concert label.

The use of the ‘folk’ attribute at this time was probably influenced by how the concept of ‘cultural policy’ was interpreted. Kjellén (1915, 11), political science professor in Gothenburg, discussed the reasons behind the First World War: ‘between the legal orders irreconcilable contrarieties can be discerned; to which should be directed a look at the great battle of cultural policies that now goes so bitterly through the world.’ Kjellén used ‘folk’ as representing the communality that builds a ‘nation’. The concept had a very positive meaning and directed the public cultural aspirations in Sweden. The SAP ambition to improve the cultural standard for underprivileged workers merged with liberal *folkbildning* efforts and conservative, i.e. Kjellénian, political philosophy regarding the formation of the ‘nation’: *Svenska Folket* (the Swedish People).

The *folkbildning* ideals were manifest in the *Riksdag* decision in 1912 on a state subsidy of 13,500 SEK (equivalent to approximately 670,000 SEK or €60,500 today) to each of the orchestra societies in Helsingborg and Gävle. The *Kungliga Musikaliska Akademien* (KMA/Royal Academy of Music) was commissioned by the *Riksdag* to issue directives for the use of the subsidies. It was quoted in *Stockholms Dagblad* on 1 December 1911:

From this point of view, the Academy would like to emphasise the importance of the folk concert programmes showing a well-balanced development according to pedagogical grounds and that, especially in the beginning, even easier-to-understand pieces of music be included in the programmes; however, useless or low standard pieces should not come into question. (NSO, 1962, 8–9)

The earliest archived HSO ‘folk’ concert programme leaflet is from 29 December 1901. It includes 16 short pieces of popular music by (near) contemporary Scandinavian composers such as Niels W. Gade and August Söderman. Also after the first *Riksdag* subsidy was granted in 1912, the first folk concert consisted of only Scandinavian composers. The focused piece was Edvard Grieg’s orchestral suite from *Peer Gynt*. Actually, the first ‘symphonic concert’ had a similar programme of Scandinavian music featuring Hugo Alfvén’s *Midsummer Vigil* (or *Swedish Rhapsody No. 1*). The management charged twice the price, 0.5 kronor (SEK), for the ‘symphonic’ concerts—probably meant to attract a more refined audience—compared to the folk concert price at 0.25 SEK. The first year included 32 ‘folk’ concerts, 13 ‘popular’ concerts, eight ‘symphonic’ concerts and 13 ‘other’ concerts. The ‘popular’ concert had a somewhat more ‘serious’ or ‘classical’ content, but compared to the ‘folk’ concert there is, generally, only a very slight difference.

The emphasis on the outreach to new audiences was paramount during the HSO’s first decades. The labelling of concerts as ‘folk’ was left from the 1934–1935 season. Alongside 10 symphonic concerts, 36 ‘popular concerts’ and 10 ‘educational’ concerts were then performed in the new hall. During the 1957–1958 season, an equal number of ‘popular’ and ‘symphonic’ concerts was performed. Thereafter, there was a rather swift move to a vast majority of ‘symphonic’ concerts.

The SFO was formed in 1902 as *Konsertföreningens Orkester* (The Orchestra of the Concert Society). Three weeks after its first ‘symphonic’ concert, a first ‘folk’ concert was offered. It consisted of contemporary Swedish pieces plus Beethoven’s 4th symphony that was also performed in the first ‘symphonic’ concert. The orchestra performed in the KMA main hall. After a temporary closure from 1910 to 1914, the orchestra was restarted as a permanent orchestra performing in the Auditorium.^2^ On the whole the SFO performed a more ‘symphonic’ repertoire than the HSO. This kind of music was also played in Sunday afternoon ‘popular symphonic matinees’. In 1914, a ticket to these Sunday concerts in the Auditorium could be bought at a 50% discount compared to the regular Thursday evening ‘symphonic’ concerts. In 1926, the Stockholm Concert Hall was opened with the SFO as its main attraction. In the 1929–1930 season, 24 ‘symphonic’ concerts were offered at a double price compared to the 30 ‘symphonic matinees’ and the six ‘popular’ concerts. Strangely, in the autumn of 1932, around the time when the HSO left the ‘folk’ concert label, the SFO started to use it instead of the ‘popular’ concert label. However, beginning in the autumn of 1940, all concerts were labelled ‘symphonic’ concerts except 10 ‘school’ concerts on Saturday afternoons intended for pupils and university students.

At this point in time the SFO was contracted also as ‘The Swedish Radio Entertainment Orchestra’, playing a lot of popular music on the public wireless service for listeners all over the country. For some other more symphonic concerts the public service radio called the orchestra ‘The Stockholm Radio Orchestra’. Most of the concerts for the public service radio were performed in the smaller Grünewald Hall within the concert house. For these radio concerts the orchestra was compensated with 30% of its total income. These concerts under the alternative names were not included in the SFO season programmes. The workload must have been considerable with, for example, 10 regular SFO concerts, two concerts as ‘The Stockholm Radio Orchestra’, and six concerts as ‘The Swedish Radio Entertainment Orchestra’ during the single month of November 1940. The Swedish Radio company soon restarted its own entertainment orchestra, but the financial contribution to the SFO remained at the same level. It reached a peak at 35% in 1956. In November that year, SFO musicians were part of ‘The Swedish Radio Symphony Orchestra’ only three times. The Swedish Radio Symphony Orchestra (SRSO) as a separate entity started in 1967. It got its own venue in 1979: the Berwald Hall.

The *Kungliga Operan* (Royal Swedish Opera) in Stockholm was started in 1773 by King Gustav III. Here we take the year for the opening of its new home in the ‘Oscarian’ opera house in 1898 as the starting point for the time series. The idea of outreach to underprivileged groups also permeated into the opera management’s programming. During the 1920s, ‘folk’ performances at reduced prices were offered and consisted of, for instance, Mozart’s *Die Zauberflöte*, *Don Giovanni* and *Le nozze di Figaro*, Puccini’s *Turandot*, Verdi’s *La forza del destino*, Wagner’s *Tannhäuser*, Strauss’s *Rosenkavalier* and Delibes’s *Lakmé* (KTA code L 3 B)*.* Actually, it was the same operas that were offered on other weekdays at double price for subscribers. A reasonable conclusion is that the kind of audience that filled the subscription performances were people who wanted to separate themselves from ‘folk’ in general and draw on the opportunity for a more elevated social identification. Today *Kungliga Operan* does not separate performances for the various population segments. It claims that it ‘welcomes all regardless of clothing style—the most important is that people can feel comfortable and be delighted’ (Sveriges Radio, 2014).

## Data

Three kinds of quantitative information were collected:

1. Data on ticket prices come from various Swedish archives: *Kungliga Biblioteket* (The Royal Library), *Riksarkivet* (RA/The Swedish National Archives), *Kungliga Teaterns Arkiv* (KTA/The Royal Swedish Opera Archives), *Regionarkivet Stockholm* (The Archives of Region Stockholm) and the city archives of Malmö and Helsingborg (*Malmö* and *Helsingborgs stadsarkiv)*. Ticket prices normally are published in season programmes or concert programme leaflets. For the *Kungliga Operan* (Royal Swedish Opera), data were collected from the opening of the new current ‘Oscarian’ opera house in 1898. Season programmes for the *Helsingborgs Symfoniorkester* (HSO^3^) were found from the opening of the new concert hall in 1932 until 1998. The orchestra management provided copies of season programmes for 1999–2020. For the *Stockholms Filharmoniska Orkester* (SFO^4^), ticket prices were collected from the *Kungliga Biblioteket*, the *Riksarkivet* and the *Regionarkiv Stockholm.* As the amount of longitudinal data are the highest for these three institutions—the Royal Opera, the HSO and the SFO—they are in focus below. However, references are also made to the Malmö Opera. Data on ticket prices were found for much shorter periods also for the Gothenburg Symphonic Orchestra (GSO) and the Malmö Symphonic Orchestra (MSO).
2. The same sources provided data on box office revenues and total income of the studied organisations. For the SFO, data for 2009–2019 were provided by *länsstyrelsen* (the Stockholm County Administrative Board).
3. For the worker wages from 1898, Svante Prado’s statistics have been used (Prado, 2010). His time series has been updated with a few years: 2008–2013 (Riksbanken, no date). Worker wages for 2014–2019 are provided online by Statistics Sweden (2020a). In order to get a time series compatible with Prado’s for earlier years, the mean hourly wages of ‘ore processors and well drillers’ were then chosen. The statistics show male worker wages.
4. This is a study based on statistics for up to 120 years. During the first few decades, the number of female workers and clerks was small, especially in comparison with the collection of data regarding male workers in the same categories. Female attendance will also be discussed below, although this analysis was based on a much less rigorous amount of data.

In Swedish official statistics during the full studied period the work force is divided into two strata: workers and clerks.^5^ For the latter category, the construction of the time series was more complicated. For most years, their salaries were recorded per month, while the data for workers were based on hourly wages. Hence, the clerk remunerations had to be re-calculated to salaries per hour. The introduction of vacation laws with increasing numbers of weeks off for all employees over time and successive Working Hours Acts had to be taken into account.^6^ Only data for clerks in the private sectors—the same as workers—were collected. For 1898–1912, railway clerk salaries were used (Bengtsson & Prado, 2019). *Socialstyrelsen* (The National Board of Health and Welfare) published salary statistics for 1913–1972. For 1913–1952, their wage statistics were issued in ‘*Lönestatistisk årsbok för Sverige*’ (Year book of wage statistics for Sweden). For 1953–1972, the *Socialstyrelse*n yearly wage statistics in ‘*Löner 19XX’* (Wages 19XX) were used. The *Socialstyrelsen* year books are provided online by Statistics Sweden. For 1973–2019, Statistics Sweden has provided the salaries for each year (Statistics Sweden, 2020b). To be comparable to the male worker wages, the male clerk salaries were used. For years that the statistics show monthly salaries, they were multiplied by 12 to get yearly salaries. The yearly amount of work hours was then calculated using the formula:

- ((52 [number of weeks per year] − number of vacation weeks according to Swedish law) * number of weekly working hours) − ((number of weekly working hours/number of days at work per week) * 10 [number of red letter days excluding Sundays]) = yearly amount of work hours
- example: for 1950 the formula gives: ((52 − 2) * 42) − ((42/6) * 10) = 2030 work hours

**Fig. 1:**
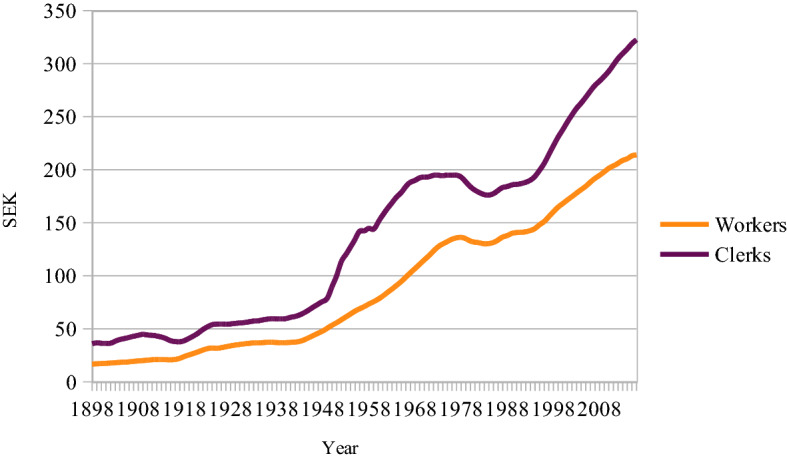
Real hourly wages for Swedish workers and clerks, 1898–2016, SEK. Base year 2020, 7-year moving average. Sources: Prado (2010), Riksbanken, Statistics Sweden (2020a), Bengtsson and Prado (2019), Year book of wage statistics for Sweden, *Socialstyrelse*n (Wages 19xx) and Statistics Sweden (2020b)

Real wages are shown in Fig. 1. The slump in the 1980s was due to a high inflation rate, with which the increase in wages did not keep pace.

Qualitative information useful for the interpretation of the quantitative data was collected through strategic interviews with management representatives from the recent past and today. From a list of the top 10 most desired respondents—not at all random—six agreed to participate. They were:

Staffan Becker: general manager of The Berwald Hall, The Swedish Radio Symphony Orchestra and Choir.
Monica Fredriksson: general manager of Folkoperan.
Bengt Hall: former general manager of The Royal Opera, the Malmö Opera and the MSO.
Åke Holmquist: former SFO general manager and permanent secretary of The Royal Academy of Music.
Helena Wessman: former general manager of GSO and the Swedish Radio Symphony Orchestra and Choir.
Fredrik Österling: general manager of HSO.

The interviewees contributed with information about the actual discussions behind closed doors. However, only minor parts of what was discussed in the interview—the items most connected to the core topic here—are rendered below. The analysis inputs will be denoted by the respondent’s initials.

During the second half of the nineteenth century, Swedish workers benefited from favourable external factors, such as globalisation forces, the prospect of mass migration to the US and a well-integrated and flexible domestic labour market. This resulted in a lack of surging skill differentials, small urban–rural wage gaps, a strong convergence of real wages across regions and, foremost, increased wages. Actually, Swedish workers had wage levels above most workers in most other European countries (Ericsson & Molinder, 2020). In the first half of the twentieth century, Swedish employees gained from strong trade unions. Johansson (2006) has analysed how average incomes increased between 1951 and 2002. He finds difficulties due to three changes in how incomes were defined and calculated by Statistics Sweden. According to his graph 6.2 (Johansson, 2006, 11) the average income, in real terms, increased fourfold for all above the age of 20 between 1951 and 2002. According to Statistics Sweden (2021) the real average income has risen by 50% 2000–2019.

**Fig. 2:**
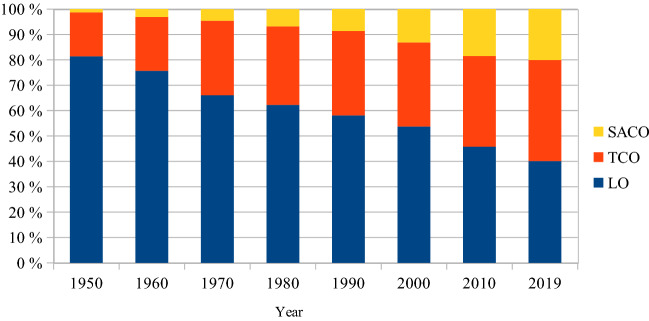
Shares of Swedish trade union memberships. Source: *Statistisk Årsbok* (Statistics Sweden’s annual book), respective years

Most of the wage increases have been negotiated collectively by trade unions, but some are ‘wage drifts’ due to individual agreements. In 1931, workers’ and clerks’ shares of their combined total number of employees were, respectively, 86.4% and 13.6% (Kommerskollegium, 1931). The restructuring after the Second World War of industry and services from manual labour to an increased demand for skilled employees has meant a lower share of workers and a higher share of clerks and professionals with academic degrees. In Fig. 2, trade union memberships for the blue-collar^7^*Landsorganisationen* (LO/The Swedish Trade Union Confederation), the white-collar *Tjänstemännens Centralorganisation* (TCO/The Swedish Confederation of Professional Employees) and *Sveriges Akademikers Centralorganisation* (SACO/the Swedish Confederation for employees with academic education) are used as proxies for the actual numbers of employees. If in the early twentieth century it was financially beneficial to attract workers to fill the halls, this is now not as important.

According to LO (2021a) the membership ratio has decreased during the last 25 years. In 1995 it was 88% for workers and 84% for clerks. In 2019 it was down to 61% for workers and 72% for clerks.

Owen (1971) and Ekelund and Ritenour (1999) find that increased wages result in increases also of the opportunity cost/price of time. People are then less inclined to spend their precious leisure time in concert halls. These authors conclude that the opportunity cost for time-intensive cultural activities will add to the negative price elasticity of ticket prices.

## Ticket prices

In the beginning of the twentieth century, public bodies started to provide grants to orchestra societies with the explicit directive that they must offer ‘folk’ concerts at low entrance fees:

Bourgeois 19th-century liberalism was aware of the broadening of classes that was going on in society. It was natural for it to use the art of music for humanitarian purposes in support of working class families. However, the folk concerts were not organised as charities but had a formative purpose. They were sometimes called ‘workers’ concerts’. . . . But it turned out that also these concerts were filled mainly with bourgeois audiences. This was identified as a problem. When in 1904/05 it granted the Concert Association in Stockholm 1200 kronor [approximately 69,000 SEK or € 6250 in 2020] for folk concerts, the Stockholm City Council explicitly demanded that the tickets be reserved for the working-class public. (Wallin, 1977, 63–64)

For the ‘folk’ or ‘popular’ concerts during the first decades of the twentieth century, the orchestras charged prices reduced to 50% of the ‘symphonic’ concerts. The programming was not radically different. For the HSO the main difference seems to have been that ‘folk’ concerts were performed in the *Folkets Hus* (People’s House), while ‘popular’ concerts were performed in the Boys’ Secondary School and ‘symphonic’ concerts in the City Theatre. The separation of concert categories was a response to public funders’ demand for inclusion of the working class. But, obviously, it also directed the social elites to the more expensive concerts where they could indulge in socialising only with people of their own kind. It followed the idea of Rosen and Rosenfield (1997) that ‘serving customers in cheap second-class seats limits the seller’s ability to extract surplus from expensive first-class seats because some switch to the lower class.... Increasing the differences in quality between classes limits substitution and allows higher prices to be charged to customers with high reservation prices.’ In this case the orchestra offered a quality difference between low- and high-class concerts through the choice of venue and the ticket price.

All studied institutions have used subscriptions as a method to secure a foreseeable future income. Normally, this meant that the subscription was limited to specific dates and productions. However, when the Malmö Opera was established in its present venue in 1944 it used a different tactic. A booklet was sold for five SEK with coupons that could be used for a purchase of two tickets for a performance on weekdays. From later data it seems that there was a discount on the ticket prices if those coupons were used. In 1960, for instance, there was a 28% discount for subscribers on weekday tickets.

**Fig. 3:**
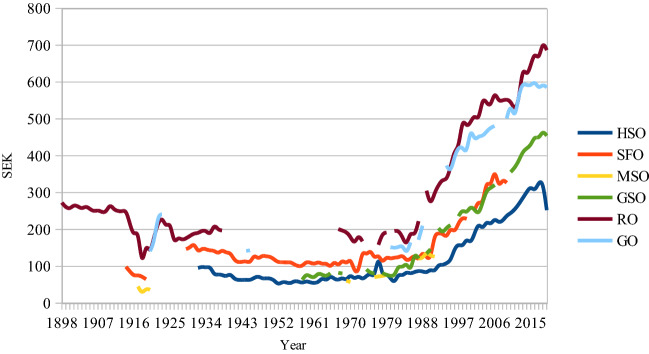
Ticket prices, Sweden, 1898–2020, CPI-adjusted, base year = 2020

For the sake of consistency, what has been recorded here for the ticket price time series are the stalls prices for ‘normal’ concerts and performances. This means that also for the years when ‘folk’ concerts and performances were offered, the time series show the development of ‘symphonic’ performances (which later became the normal concert category). In 1898 this normal price at the Royal Opera was 4.5 SEK—now it is 820 SEK (approximately €76). When the SFO was restarted in 1914, a ticket to the symphonic concerts cost 2 SEK—now it is 225–340 SEK. When the HSO gave its first concert in its new venue in 1932, the normal ticket price was 3 SEK—now it is 250 SEK. Figure 3 shows CPI-adjusted ticket prices.

Presently opera and concert hall managements perceive the content of performances as much more decisive than ticket price levels for whether they will succeed in reaching a new and broader audience (SB, ÅH). The format choice, for instance a ‘festival’ presenting a single composer or the use of a ‘soup’ or ‘coffee’ concept with food and beverage added to the music, is also crucial (ÅH). All respondents declare the present general level of ticket prices as historically given, as the only or main discussion is on how much they can be raised. This represents a clear case of ‘path dependence’. Price discrimination set-ups are used uniformly regardless of content.

The Malmö Opera seems to be the institution that has experimented most with price discrimination. Its experience from setting a very low price, 100 SEK, for some seats in the rear of the venue and along walls for all performances during the 2010s was that they were the least preferred choice by most ticket purchasers. The most expensive tickets were sold first (BH).

The SFO general manager during the 1986–1999 period claims that ticket prices were not, during his time in charge, a decisive factor for the ticket demand (ÅH). Scheff (1999) finds the same survey response from arts marketing managers in the USA. Although this may have been true for the SFO during the 1990s, it is clear from the acquired statistics that in previous decades there was a negative price elasticity in some instances. When in 1959 ticket prices to SFO concerts were increased by 8.7% (*Riksarkivet* code L 1:19–20), the mean audience number per concert decreased by 3.0%. In 1970 ticket prices were raised by 10.7% with a 3.7% decline in audience numbers (*Kungliga biblioteket*, section ‘*vardagstryck*’, card no. 18663). Ekelund and Ritenour (1999) find a similar negative price elasticity regarding the demand for symphony orchestra tickets in the USA from 1973 to 1992. For HSO the same pattern occurred in the first decade of the new millennium. The sale of tickets (excluding subscriptions) fell by 30% (*Helsingborgs stadsarkiv*, code B 1:1) when the ticket prices were raised by 5.3% in 2008 (season programme copies obtained from the HSO management).

The price elasticity findings presented by Heilbrun and Gray (2001) mostly come from the US musical scene. However, the price inelasticity that Heilbrun and Gray report is probably also relevant in the Swedish context. Heilbrun and Gray suggest that institutions’ management may set ticket prices too low, ‘for if demand is price-inelastic, attendance will not fall very much if ticket prices are raised, and total revenue will increase substantially’ (Heilbrun & Gray, 2001, 103). It seems that the institutions studied here have followed this advice and raised their ticket prices without experiencing declining audience numbers. However, in that process, they may have had to rely on the more well-to-do part of the society as their audience.

According to the audience statistics in the annual reports and the numbers of total annual visitors from the historical archive on its website, the SFO reached a mean concert audience number of 1118 during 2019. In 1947 the average concert attendance peaked at 2010. However, after major acoustics-enhancing measures in the early 1970s, the full capacity of the main hall is now reduced to 1770. In the last year before the rebuilding, the mean attendance was down to 1433. The competition from other sources of leisure activities had grown fiercer than in the peak year 23 years earlier, and it is today, another 50 years later, even harder. Hence, it is more difficult for a symphony orchestra to attract ticket buyers. The high ticket price policies have not made it easier. In 1970 the average worker would have had to work 1 h and seven minutes to be able to buy a ticket to an SFO concert, while in 2019 the necessary workload was up by 40 min. If all the 1,118 visitors in 2019 paid the 325 SEK stalls ticket price, the mean concert income would have been 363,350 SEK. For the same total box office income from a full-capacity audience it would suffice with a ticket price of 205 SEK. As ticket price policies are path dependent and uniform, such pricing experiments do not occur.

Sometime during the late 1980s, the SFO management calculated the financial effects of free admission to its concerts. Then costs for marketing and box office staff could have been kept extremely low. The calculus produced almost no negative effect. However, the idea was not tested and, instead, eventually a marketing officer was recruited (ÅH).

All respondents claim a close contact with the box office personnel and audience hosts. They are the first-line communicators with the audience. Audience comments on the artistic content and quality are rare. The same goes for ticket prices, as those who attend obviously have the financial means (MF). Comments instead revolve around topics such as lack of parking possibilities (ÅH), slow café service and long lines to the ladies’ rooms. One respondent used to spend time in the vicinity of the box office an hour before concerts to pick up on audience reactions (ÅH).

The experience of a few of the respondents is that ticket prices are formally decided by boards of directors on suggestions from steering committees in which marketing directors are members. However, in some institutions this decision is in the hands of the managing director.

As shown in Fig. 3, CPI-adjusted ticket prices have increased substantially since the mid-1980s. It seems from the annual financial reports that raised ticket prices were necessary to cover the increased costs. However, one cannot rule out the possibility of the opposite causality: increased costs were made possible by the increased box office revenues. We now turn to the main question in this study: has this been detrimental to workers’ access to opera houses and concert halls?

## The workers’ penalty

**Fig. 4:**
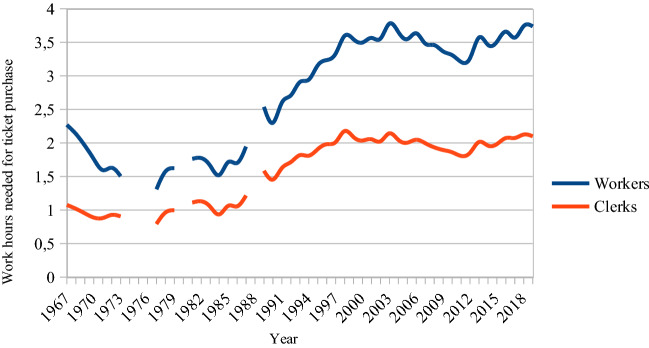
Average hours of paid income needed for Swedish male workers and clerks to buy a ticket to the Royal Opera, 1967–2019. *Arbetare* (workers) and *tjänstemän* (clerks)

When the Royal Opera’s current house was opened in 1898, workers’ average hourly income was 0.28 SEK (or CPI-adjusted with 2020 as the base year: 15.75 SEK). For that he or she could buy 9 kilos of butter (Edvinsson & Söderberg, 2008). The equivalent amount that a worker could buy for 1 h of work in 2019, with mean income at 182 SEK per hour, is approximately two kilos of butter. Even if the purchasing power in the food market in 1898 were substantial, the worker, nevertheless, had to spend 16 h at work to be able to buy a ticket to the Royal Opera. In 2019 it was down to 4 h and 40 min. During the first decade after the 1974 CPA, the worker needed only one and a half hour of work to be able to afford the opera ticket. In Fig. 4, the workers ‘penalty’ versus that of clerks is seen visually.

**Fig. 5:**
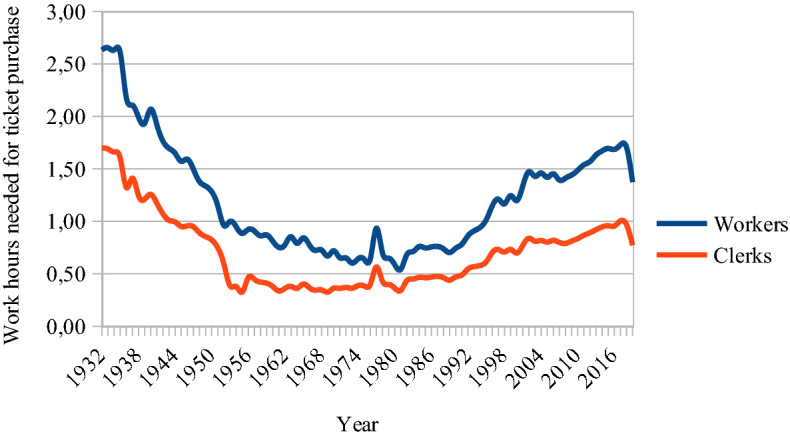
Average hours of paid income needed for Swedish male workers and clerks to buy a ticket to the HSO, 1932–2019

Figure 5 presents similar data regarding the HSO but for a longer period. In 1932, the worker had to work an extra hour compared to the clerk. While in 2019 it took an industrial worker 1 h and 22 min of work to buy a ticket to the HSO, the clerk was able to do so for 36 min less. This ‘workers’ penalty’ in Helsingborg was at its lowest during the first 2 decades after the 1974 Cultural Policy Act. During that period the CPI-adjusted ticket price was approximately a quarter of the 2019 ticket price. This, of course, decreased the worker’s penalty. Now the penalty is at a level similar to the 1940s. However, for a ticket to the then-frequent ‘popular’ concerts with reduced prices, the penalty was approximately 20 min lower.

**Fig. 6:**
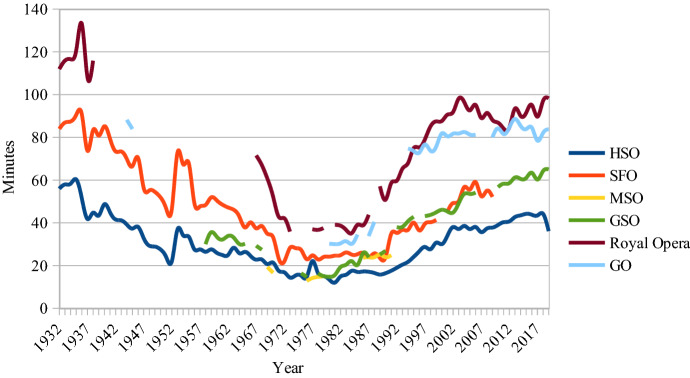
Worker’s penalty, Sweden, 1932–2019. (Average yearly wages)

For clarity reasons only the workers’ penalties—the gaps between the lines in Figs. 4 and 5—are shown in Fig. 6 where data from all the studied institutions are presented. How many more minutes does the average worker have to work to also be able to buy a ticket?

For an SFO ticket the worker now has to spend 47 min more at work than the clerk. This is the same disadvantage as in the early 1960s. The worker was the least disadvantaged during the 1970s and 1980s when the penalty was halved.

It is obvious that the concern for low-income audiences was strongest in the years before and after the 1974 CPA. Although there are still examples of price discrimination policies with lower-priced tickets for some individuals, for some days of the week and for some seats, the general conclusion must be that managements now focus more on maximum proceeds from those used to going to the opera and the concert hall, i.e. those showing high demand, and those who are better off. The goal for social inclusion in general and the inclusion of workers in particular seems to be of decreasing importance when ticket prices are decided.

**Fig. 7:**
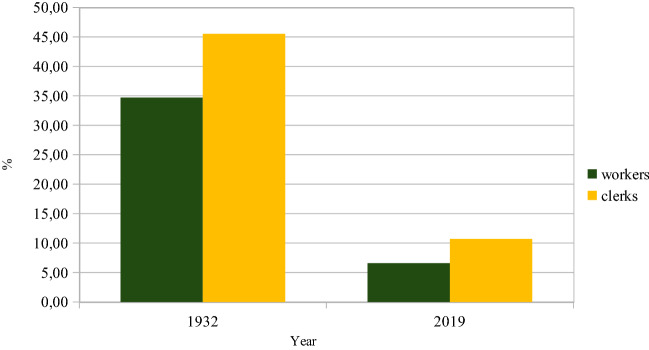
Income gaps for female versus male workers and clerks, 1932 and 2019

Our focus is on male workers and clerks as the actual numbers of female workers and clerks were low at the beginning of the studied period. The income gaps for females in 1932 and 2019 are shown in Fig. 7.

Clearly, female workers and clerks had to spend more time than their male colleagues to be able to pay for a ticket. In 1932, the share of housewives was higher than in 2019, so the husband’s income then had to cover two tickets if both were concert or operagoers. In 2019, almost 80% of women earned their own income. For couples, now two incomes are available for ticket purchase. Unfortunately, statistics on the sex of audience members are rare in Sweden. A 1960 Royal Opera survey showed that women constituted 64% of their audience (KTA code H 4: Audience survey, 1960, table PU 2). In 2020, 13% of women in Sweden attended a classical concert or an opera performance at least once compared to only 10% of men. For rock concerts, male attendance reached 23%, while female attendance was lower at 19% (Kulturanalys, 2021, Table 4). Of course, percentages were lower this pandemic year than in prior years. For both classical concerts/opera and rock, there was a stark overrepresentation of visitors with a high level of education. It is likely that the higher percentages for people with high education also have elements of an income privilege. In terms of age, the only main difference between classical/opera and rock concerts was the reluctance among the 65–85 age group to attend the latter. For other age categories, the attendance rates were surprisingly equal for both classical/opera and rock concerts. Although men are less likely to attend cultural activities, those who do attend seem to, according to Ateca-Amestoy (2008), do it slightly more frequently than women. However, due to the structure of the Swedish data, that finding cannot be corroborated here.

## The share of box office revenues in total income

**Fig. 8:**
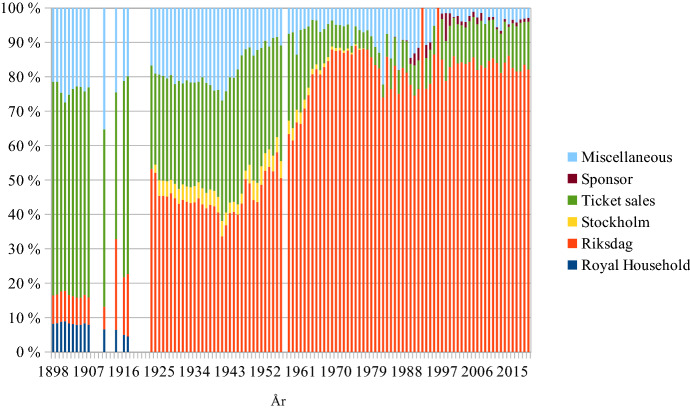
Shares of total income, Swedish Royal Opera, 1898–2019

In the early decades of the Royal Opera box office incomes, *‘recettes*’ were marginal. King Gustav IV Adolf contributed 94% of the total income in 1803 (KTA, code G1 vol. 4). In 1860 King Karl XV paid for 20.3% of total costs, the *Riksdag* covered a further 37.3%, rents 12.5% and *recettes* 29.5% of total costs (KTA, code G 4 A). When the Oscarian opera house opened in 1898, the *recettes* peaked at 62.1% of total income. In 1982, at 3.8%, they were even lower than 1803 (see Fig. 8) (KTA, code L 3 A). According to the Royal Opera annual report for 2019, ticket sales were then again up: 14.0%.

**Fig. 9:**
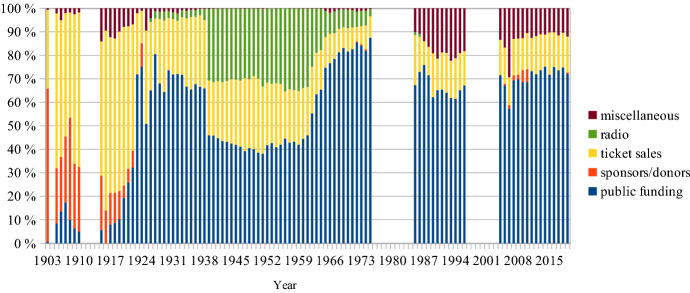
Shares of total income, SFO, 1903–2019

When the SFO was restarted in the autumn of 1914, it had no royal or state funding. The City of Stockholm contributed 5.4% of total income and donors made up another 21.9%. Ticket sales brought in 54.0% of total revenues (Riksarkivet, code 730139/B 4:1). It reached its lowest share, at 3.3%, in 1972 when the 1974 CPA was discussed and anticipated (Regionarkivet, code LA_579-1/G1:1). Since 1990 ticket sales have revolved around 10% (see Fig. 9).^8^

**Fig. 10:**
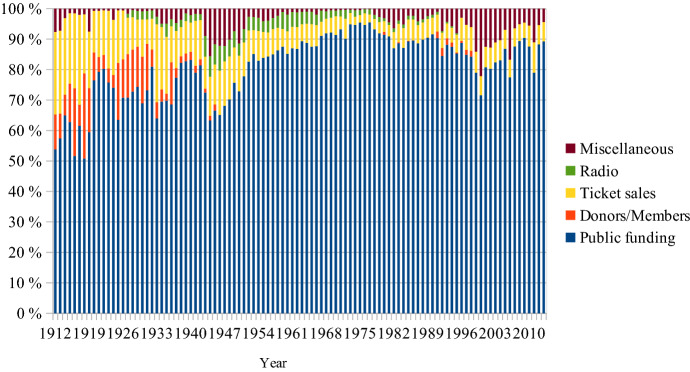
Shares of total income, HSO, 1912–2013

For the HSO public funding has been and still is even more important than for the Stockholm institutions. It peaked in 1975 with 94.7% of total income. Ticket sales then reached an all-time low at 2.5% (see Fig. 10) (Helsingborgs stadsarkiv, code 151). Beginning in 2014 the concert hall and its orchestra became part of the Helsingborg Arena group. The annual reports of this parent company are generalised and do not offer sufficient granularity to be relevant for this study.

If box office revenues were the primary source of income a century ago, they are now of almost marginal financial importance. A spontaneous thought then could be that this marginal importance will make price increases rather pointless, especially in view of the explicit CPA demands for social inclusion of the underprivileged. All respondents, however, regard the level of box office income as absolutely crucial. It is claimed that they contribute to the current high-performance quality. The ticket price must remain at the same or a higher level where the institutions keep their ‘high status’ reputation (HW). The price is a token of ‘value’: high price = high value (SB, MF, HW).^9^ The tangible object is a receipt indicating the ticket holder’s self-identification (FÖ). Pierre Bourdieu (1979, part III:8) relativises this lingering idea of high status based on a ‘habitus’ of a ‘class’ of people who have—or at least claim to have—the capacity to enjoy ‘fine arts’. Many authors have since followed in Bourdieu’s footsteps. David Hesmondhalgh (2013, 52), for instance, finds that musical ‘omnivorousness has replaced snobbery as the goal of “highbrow” taste’. Bjurström (2018) concludes that Swedish national music policy has become ‘post-aesthetic’:

… [it] shows growing reluctance to interfere in issues or negotiations on aesthetic values and qualities. At the same time, one may ask to what extent it, like public cultural policy in general, still tacitly leans towards an educational perspective in its positions on the intrinsic value of culture, a high-quality cultural offer and actual support for various types of cultural activities, a ‘Bildung’ perspective that it may have left behind but not explicitly renounced.

David Throsby (2001, 27–29) claims that postmodernism since the 1980s, ‘while focusing attention on an expanded view of [cultural] value, has failed to offer a satisfactory account of how value might instead be perceived and evaluated’. He suggests a mapping system in which the aesthetic value is but one of six separate value concepts, the others being the spiritual, social, historical, symbolic and authenticity values. Throsby, together with Michael Hutter, has since expanded the discourse around the one-dimensional economic value on the one side and the multi-faceted cultural value on the other (Hutter & Throsby, 2008). Hence, the notion of high status is not a universal truth. It is refuted by many. However, for those who decide on ticket prices in Swedish opera houses and concert halls, this perception of the high status of what is offered is crucial.

Some respondents claim that public funders now put extra pressure on them to increase their self-financing (SB, MF, FÖ). Funders obviously treat performing arts institutions differently than museums and libraries, for which they demand free admission, although the same CPA objectives apply for all cultural policy actors. The performing arts institutions were included in the ‘entertainment tax’^10^ system of 1919–1963 (Albinsson, 2021). One possible reason may be that there is a lingering view on the performing arts as ‘entertainment’ rather than ‘culture’ in a stricter sense. Swedish newspapers still publish opera and concert reviews on the pages for ‘entertainment’, whereas, for instance, literature and museum exhibition reviews are filed under ‘culture’.

A common analysis among the respondents is that public funding has not increased at the same rate as costs. Hence, a subsequent rise of ticket prices has been necessary. However, rising costs have exceeded inflation. For the HSO the public funding increased yearly by an average of 6.3% between 1990 and 2013, while the CPI increased by a yearly average of only 1.8%. The ticket price was raised yearly by 7.1% on average. The SFO received increased public funding at an average yearly rate of 4.0% from 2004 to 2019, while the CPI increased with a yearly average of a mere 1.2%. Finally, the Royal Opera’s grant from the *Riksdag* increased on average by 2.9% yearly between 1997 and 2019, while the CPI increase had a yearly average rate of only 1.2%. The price of the Royal Opera tickets was raised by 4.4% per year on average. So the rise in ticket prices, if necessary at all, would only cover a within-business increase of costs higher than the inflation rate.

Baumol and Bowen (1966) describe the productivity problems of the performing arts in the USA in the decades before 1960, writing that the same problems of replacing people with machines exist in other parts of the knowledge-intensive sectors of society such as health care and education. Baumol returns to this ‘cost disease’ issue half a century later:

[T]he cost disease has brought profound changes in the way we live. If it continues to influence the workings of the economy, the consequences may be even more far-reaching. With continued growth in general productivity, the typical household may enjoy an abundance of goods, but if governmental responses are poorly considered, citizens also may suffer from great deterioration in public services such as garbage removal. The services of doctors, teachers, and police officers may become more automated and impersonal, and the arts and crafts may be increasingly supplied only by amateurs—the cost of professional work in these fields may be too high. In these circumstances, people may begin to question whether the explosion of the supply of material goods has really improved their quality of life. (Baumol, 2012, 43–44)

The ‘cost disease’ may, at least to some extent, be counteracted by economic growth in other sectors. This provides increasing tax revenues that can be used to mitigate the effects of lower productivity growth in knowledge-intensive public services. Tax revenue growth has enabled public contributions to culture higher than the rate of inflation. This explains the rather rapid increase in public funding. However, it does not necessarily explain the equally rapid rise in ticket prices. The balance between costs and revenues, and thus the level of turnover, is a decisive factor for which the institutions are, themselves, responsible. If public funders put pressure on managements to increase their self-financing, this can be seen as the former’s response to the latter’s choice of accepted cost levels.

## The current directive for social inclusion

Figure 2 shows how the labour market has changed from an intense blue-collar dominance to a much higher degree of skilled labour. Venues can now be filled with only white-collar employees. Contrary to a century ago, there is today no financial necessity to attract the working class. However, the number of workers in Sweden is now at least three times higher than it was at the beginning of the twentieth century when the cultural policy focus was so explicitly on them (see Fig. 9). So if the working class today opts out of these types of cultural experiences, it is the choice of many more individuals than before.

**Fig. 11:**
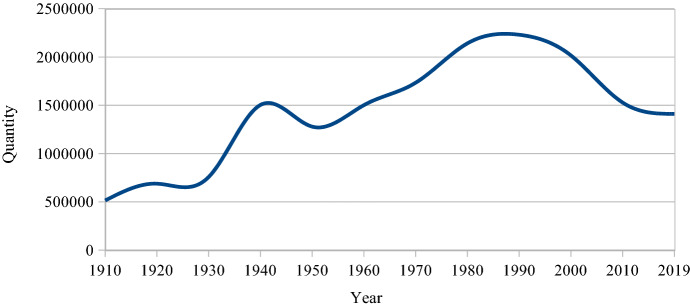
Number of workers, Sweden, 1910–2019. Sources: 1910–1950: *Statistisk årsbok*, 1950–2019: LO membership

Hence, the social inclusion issue has increased in importance. However, during the last three decades, the total number of workers has declined. New technologies have been introduced and to some extent, they have replaced manual labour. As seen in Fig. 11, the decline has levelled off during the 2010s. This may possibly be due to the fact that the numbers of welfare workers and employees in the retail business have increased. Employees in these occupations now make up 47.4% of the LO membership numbers compared to 43.2% 10 years ago (LO, 2021b). This share will probably rise. The number of workers in new kinds of low-income occupations will most likely increase. How this will influence the future numbers of low-income workers is hard to predict. One crucial current factor is how unskilled immigrants and refugees will be included in the future labour force to a higher degree than presently.

As the willingness to pay membership fees to trade unions has decreased, the actual number of workers is now higher than what is shown by the LO membership indicator. In the middle of the 1990s, 88% of workers were members of unions. Before 2007, tax deductions for trade union membership fees could be made. This possibility was removed by the then new liberal-conservative *Riksdag* majority, and it has not been re-introduced by the social democratic minority government in position from 2014. In 2020, LO (2020) reported that 61% of workers were members of trade unions (72% for clerks). The willingness to pay membership fees was also higher for women (64%) than for men (59%). In terms of age, it appears that as workers get older, their willingness to pay membership fees increases: for the 45–64 age group the union accession was 72%, while only 39% of the workers in the 16–24 age group paid the dues.

The ticket price statistics above indicate a contemporary lack of interest among managements to cater to the interests of the working class, specifically, through the use of the pricing instrument. Since 1990 ticket prices have risen much more than wages. Furthermore, the income gap between workers and clerks has increased (see Fig. 1). The 1974 CPA puts the needs of ‘disadvantaged groups’ in the forefront. In 2009, the Cultural Committee Report suggested a communitarian goal in the future CPA: ‘reinforce and further an open social community’ (SOU, 2009:16, 21). The government’s bill for the 2009 CPA revision reformulated this into a more individualistic objective that public cultural institutions shall ‘promote everybody’s possibilities for cultural experiences’ (Proposition, 2009, 26). This kind of objective is now part of the management DNA (HW). Social inclusion is a national cultural policy ideal, and it permeates into regional and local cultural programmes which often have elaborate descriptions of what is desired. For instance, the new 2020–2023 Cultural Plan for Region Västra Götaland^11^ has as a general objective using all the current political buzzwords—that ‘culture in Västra Götaland must be based on everyone’s right to participate in culture activities regardless of background, gender identity, ethnicity, religion, socioeconomic status, place of residence, age, sexual orientation or disability.’ When it comes to the performing arts in particular, the explicit demand is that ‘To care for the existing audience and to find new audience groups the performing arts need to be more accessible while the artistic expression remains powerful’ (Region Västra Götaland, 2019, 22). Criticism regarding the tendency to use cultural institutions as instruments for other objectives than ‘cultural’ in a more strict sense is recognised in the new cultural policy programme for the City of Uppsala: ‘Culture can contribute to innovation, jobs, economic growth, integration and security. There is a risk that culture is reduced to having only instrumental significance. Culture then becomes a means to achieve other purposes than the cultural experience itself.’ Yet the Uppsala programme, furthermore, declares that culture ‘can also increase social cohesion. In a culturally sustainable society, the living environment is attractive and the city’s character and history are pronounced’ (Uppsala, 2020, 2–4).

The studied cities all show a substantial population growth during the period studied here. The number of administrative cities, towns and municipalities in Sweden has been successively reduced. As a consequence, Stockholm, Malmö and Helsingborg have grown due to the incorporation of suburban municipalities and from the influx of people from rural areas and of immigrants. With these conditions both the SFO (1929–1971) and the HSO (1932–1999) show a positive influence on the mean audience from the growth of the population. However, the Stockholm population grew by 48% from 1929 to 1971 and the Helsingborg population more than doubled between 1932 and 1999. As the population grew, the share of the Stockholm population that frequented the SFO concerts was much reduced as the mean audience numbers in 1929 and 1971, respectively, were the same. The population growth of Helsingborg is evident in the mean audience numbers, which grew by 31.2% from 1933 to 1999. However, in both cases the actual number of inhabitants that did not attend became increasingly higher. Population numbers have also increased in both cities thereafter. By 2019 the Stockholm population had grown by 94% since 1930 and the Helsingborg population had grown by 164%. Most likely the shares of the populations that do not attend the SFO and the HSO concerts are higher than ever.

Lately, the ‘social inclusion’ concept of the early twenty-first century has often been interpreted as social cohesion or social sustainability. This brings a kind of present-day updated motivation for the inclusion objective which in 1911 the KMA expressed as this: ‘make precious music available to manual workers and the underprivileged’. Hence, it is also a kind of reification of the ideas behind the ‘folk’ concert.

A century ago the public financial support of the then new symphony orchestras was a response to the evolution of Sweden from an agricultural country to a modern, industrialised society. It was regarded as important to include the working class in the efforts to raise everybody’s economic and cultural standard, and the symphony and the opera were regarded as preferred means. Something similar is found in the current El Sistema music-education programme in low-income suburbs of Stockholm. The idea is that the children shall come into close contact with a kind of music which traditionally has been regarded as ‘high-status’ cultural capital. Participation in the orchestra provides the young players with an opportunity to develop in a professional trade—to identify as ‘musicians’ (HW).

Despite the fact that such a policy counteracts the social outreach mission (MF), there is, with some exceptions for the *Folkoperan*, deeply rooted support among the respondents for a high price on tickets.^12^ This is in stark contrast with, for instance, the Detroit Symphony Orchestra (DSO) with its Swedish vice president and general manager, Erik Rönmark. Following severe financial difficulties 10 years ago, the DSO reinvented itself as ‘the most accessible orchestra on the planet’:

Making the tickets more affordable, the orchestra reduced the average price to as low as that of the 1999 season, with half of the seats being $25 or less. It also came up with a tiered ticket pricing strategy to accommodate a broad range of customer demographics, including discounted tickets for young professionals ($10 per ticket with $40 annual membership fee), rush tickets for Detroit residents ($15 per ticket), and a very low-cost Soundcard student membership (free tickets to most concerts with $25 annual membership fee). (Chucherdwatanasak, 2020)

For the DSO, 2019 was the seventh consecutive year with an operational surplus and the fifth consecutive year of ticket revenue growth. The orchestra’s funding primarily comes from sponsors and donors. Ticket revenues made up 26% of total income in 2019. Part of the reinvented strategy was that the DSO’s role was ‘to be both a nonprofit cultural organization, as well as a social-service institution’ (Chucherdwatanasak, 2020). Many of the activities that the DSO is now involved in are similar to what is found in Sweden, as well. However, here they are rare among symphony orchestras but common in the ‘county music organisations’ with smaller professional ensembles of various kinds.

The substantially state-funded (82.1%) Royal Opera has a price for a stalls ticket at the same level as the commercial ‘musical’ venue *Oscarsteatern* in Stockholm that operates without public funding.

An aggravating circumstance for Swedish performing arts institutions is that blue-collar workers, and, for that matter, also many white-collar workers and academics do not necessarily seek the elevated musical experience that opera and symphony can bring. The construction of the 1912 policy regarding what was ‘valuable music’ is not as relevant today (FÖ). The 1974 CPA (and the 1996 revision) had as one of its objectives that cultural policy should ‘counteract the negative effects of commercialism in the field of culture’. This came at the exact final tipping point when commercial music made a game-changing breakthrough. Six weeks before the *Riksdag* decided on the national cultural policy goals, ABBA had won the Eurovision Song Contest and Blue Swede’s^13^ cover of B.J. Thomas’s ‘Hooked on a Feeling’ topped the US Billboard’s Top 100—both on the same day: 6 April 1974. These developments were followed by a wide range of pop groups and pop music producers with global renown. This led the 2009 revision of the national CPA to state that ‘there is no given contradiction between commercial viability and artistic quality or freedom’ (Proposition, 2009, 28), and the previous ‘counteract commercialism’ idea was left.

Obviously opera houses and symphony orchestras now operate in a completely different society with new cultural conditions. The old institutions must find new modus operandi—they need to tear down the walls of the political framework that constructed the opera and symphony ‘cathedrals’ (FÖ). ‘Thresholds must be lowered’ (SB, MF, ÅH). Programming of the opera houses and the orchestras has changed. Concerts for schools are imperative for all orchestras. The Swedish Radio Symphony Orchestra has successfully tried concerts with film music, disco music and Persian music (SB). *Folkoperan*, the ‘second’, freelance opera house in Stockholm with an explicit goal to make opera accessible for all, has cooperated with the local contemporary circus ensemble *Cirkus Cirkör* and with musicians residing in immigrant-dense neighbourhoods and specialising in music from their previous home countries (MF). The HSO works hard to modernise its concerts by, for instance, including more music by female composers and more contemporary music. This has led to a drift from subscription sales to single concert ticket sales, yet the total number of occupied seats remains the same (FÖ). The Malmö Opera presents one ‘musical’ production each season—the contemporary version of its old operetta tradition. A substantial part of the musical audience also buys opera tickets (BH). The Gothenburg Opera uses part of the proceeds from its yearly ‘musical’ show to finance opera productions.

Concert halls are now let to commercial music producers in a way unseen only a few decades ago. These events attract other audience groups than frequent the symphony orchestra concerts. However, if this represents support for the actual institutional social inclusion ambitions there should be a flow of visitors from such events to symphonic concerts. This has not been the case, and the renting out of venues is predominantly only of financial importance (ÅH).

The social inclusion ambition must remain in the future—it should never be given up (BH, ÅH, HW). Although the mental distance required for average citizens to appreciate Beethoven, for instance, could increase (SB), his music will remain a part of the global canon (SB, BH, ÅH, HW). Educational activities will be just as important. However, one should accept that the opera and symphony audience will, just as in the past, largely be grey-haired also in the future (MF, ÅH). Given the cost disease described by Baumol, which also affects health care, schools and elderly care—the current foremost political concerns—it is likely that opera companies and symphony orchestras will have to face an even harder battle for public financial support in the future (BH). Perhaps the global climate crisis will make people more interested in self-realisation through cultural experiences. A reasonable guess is that they will want to travel more within than geographically (HW).

## Conclusions

Ever since their early years more than a century ago, Swedish opera houses and symphony orchestras have operated with a specific goal to include workers, the underprivileged and the disadvantaged. The current explicit interpretation of the same ambition is to strive for ‘social inclusion’. It is likely that, in the near future, this will be reinterpreted as ‘social sustainability’ and ‘social cohesion’.

The focus of this paper is to determine how CPAs have influenced pricing policies in operas and concert halls. Clearly, they have not. Swedish opera and symphony orchestra managements do use pricing discrimination with, for instance, lower ticket prices for children and youth, for some venue sections or for some days of the week. However, ticket price levels were most advantageous during the decades around the *Riksdag* decision on the 1974 CPA when, for instance, in 1977 the average worker had to work only 1 h and 19 min to be able to pay for a ticket to the Royal Opera. Since the 1990s ticket prices have increased to a level at which audience members, workers in particular, must spend as much time at work as in the 1950s (the 1930s for the HSO). Now the average worker needs to work 3 h and 45 min to reach the 2019 Royal Opera ticket price level. For the symphony orchestra tickets the difference is less but still significant. Now twice the workload is needed for a concert hall admittance compared to the 1970s and 1980s. As the population has grown, the successive ticket price increases, in order to safeguard a ‘high-status’ aspiration, have resulted in a situation where only the better off or the most motivated and accustomed to the music genres in question attend the performances.

Swedish opera companies and symphony orchestras are heavily subsidised. The box office revenues are of marginal importance for their survival. Nevertheless, respondents believe that their ticket prices are tokens of ‘value’ and that a high price indicates the level of quality. A common analysis among the respondents is that public funding has not increased at the same rate as costs. Hence, a subsequent rise of ticket prices has been necessary. However, public subsidies have increased more than inflation but not enough to cover the within-business cost increases accepted by managements. They believe that they have been forced to raise ticket prices to maintain the conceived value of their activities.

Of course, the competition from other sources of cultural experiences has grown fiercer. Although the social inclusion objectives are included in cultural policies at all political levels, the attitude toward the commercial music industry has changed. A century ago a Social Democratic Minister of Culture considered popular music as ‘nonsense’ (Engberg, 1921). Four decades later, the Minister of Culture characterised pop music as equivalent to ‘weekly tabloids, kitschy paintings, films of dubious value and all kinds of “entertainment”’ (Edenman, 1959). The 2009 national CPA, on the other hand, claims that ‘there is no given contradiction between commercial viability and artistic quality.’ The way publicly funded music institutions try to appeal to new audience groups is through the use of content from a fundamentally commercial domain in ‘special events’. The expectation is then that this will make these audience members interested also in the standard repertoire and ‘lower the thresholds’ to the venues. The choice of a high level of ticket prices for the traditional content makes this hope in vain. The standard audience remains monocultural. The choice of ever higher ticket price levels since the 1970s counteracts the explicit goals from public funders for social inclusion.

## Data Availability

The data set will be provided on demand.

## Footnotes

1 All translations by the author.

2 The Auditorium was built in 1912 for the Stockholm Olympics within the housing body of a former gasholder. It had a capacity of 1751 seats. Beginning in 1914 it not only held the SFO concerts but also functioned as a cinema. In 1977 it was bought by the LO. It was demolished and gave way to an office building.

3 Until 1971: ‘*Nordvästra Skånes Orkesterförening*’ (The North Western Scania Orchestra Society).

4 Although the orchestra since 1992 has called itself *Kungliga*, i.e. ‘Royal’ instead of ‘Stockholm’, the acronym SFO will be used throughout for consistency.

5 *Arbetare* (workers) and *tjänstemän* (clerks).

6 Sweden got its first legal regulation regarding vacation in 1938. It stipulated a minimum of 2 weeks for everybody. Prior to that the *Yrkesfarelagen* (Law on Occupational Hazards) recommended employers to grant at least 4 days of vacation (Högberg 2008). The number of vacation weeks grew step-wise to the current five from 1990. Regarding work hours before the 1919 Law on Work Hours, a normal, negotiated maximum number of hours of work was ten per day, i.e. a total of 60 h per week (Spross 2017, 42). The 1919 law specified a maximum of 8 h. This number still prevails. However, for clerks the weekly number of work hours was negotiated step-wise so that it already from 1953 seems to have been 40. In 1971 workers were also granted this through the new law providing 5 days of work per week.

7 With the growth of the welfare sector the ‘blue-collar’ tag has become less relevant, as the LO now also includes approximately 27,000 ‘welfare workers’ in the suborganisation *Kommunal* (the Swedish Municipal Workers’ Union). They include care assistants for the elderly, bus drivers, occupational therapy assistants, kindergarten nannies, housing supporters for the disabled, meal service personnel in schools, and many more.

8 Sponsor income seems to have been filed as ‘miscellaneous’ during the 1980s. At that time there was substantial support from an insurance company, Trygg-Hansa, and a pharmaceutical company, Astra (ÅH).

9 In a previous study of small Scandinavian opera enterprises, this author found a correlation between a high ticket price and longer final applause (Albinsson 2019).

10 Swedish: *nöjesskatt.*

11 https://www.vgregion.se/en; https://en.wikipedia.org/wiki/Västra_Götaland_County.

12 The opera houses differentiate ticket prices depending on weekday/weekend and venue sections (MF, BH).

13 Swedish name: *Blåblus.*
